# Increased ischemia-modified albumin levels in children with steroid-sensitive nephrotic syndrome

**DOI:** 10.12669/pjms.36.7.2924

**Published:** 2020

**Authors:** Gokhan Cakirca, Ahmet Guzelcicek, Kenan Yilmaz, Cemal Nas

**Affiliations:** 1 Gokhan Cakirca, Department of Biochemistry, Sanliurfa Mehmet Akif Inan Training and Research Hospital, Sanliurfa, Turkey; 2 Ahmet Guzelcicek, Department of Pediatrics, Faculty of Medicine, Harran University, Sanliurfa, Turkey; 3 Kenan Yilmaz, Department of Pediatric Nephrology, Sanliurfa Training and Research Hospital, Sanliurfa, Turkey; 4 Cemal Nas, Department of Biochemistry, Sanliurfa Training and Research Hospital, Sanliurfa, Turkey

**Keywords:** Ischemia modified albumin, Oxidative stress, Steroid-sensitive nephrotic syndrome

## Abstract

**Objective:**

Growing evidence shows that oxidative stress plays an important role in the development and progression of nephrotic syndrome (NS). In this study, we aimed to examine serum IMA levels as an indicator of oxidative stress in children with steroid-sensitive NS (SSNS) in remission and relapse.

**Methods:**

This cross-sectional study was carried out at the Pediatric Nephrology Unit of Sanliurfa Training and Research Hospital, Sanliurfa, Turkey, from April 2019 to December 2019. In this study Serum IMA and albumin levels were determined in 70 children with SSNS and 45 healthy controls. Among the children with SSNS, 50 were in remission and 20 were in relapse. Then, adjusted IMA levels were calculated from the IMA/albumin ratio.

**Results:**

IMA and adjusted IMA levels significantly increased and albumin significantly decreased in children with SSNS in relapse and remission compared with those of the healthy controls. Moreover, these alterations were more prominent in the relapse group than in the remission group. IMA was inversely correlated with albumin in children with SSNS (r= −0.881, p= <0.001).

**Conclusions:**

Our findings demonstrated that elevated IMA and adjusted IMA levels observed in patients with SSNS were associated with increased oxidative stress and could indirectly reflect the degree of oxidative damage in glomerular structures.

## INTRODUCTION

Nephrotic syndrome (NS) is a kidney disease with an estimated incidence of 1.15–16.9 per 100,000 children. The main manifestations of this disease include severe proteinuria, hypoalbuminemia, edema, and lipid abnormalities. The pathogenesis of NS is not fully known, but evidence suggests that it may be related to immunological mechanisms, podocyte-related factors, and genetic variants.^1^ Some studies support the hypothesis that oxidative stress plays a crucial role in the development and progression of various kidney diseases, including NS.^2^-^4^ Oxidative stress, which results from the imbalance between the production of reactive oxygen species (ROS) and their elimination by antioxidants, can cause undesirable effects on cellular structures.^5^ Increased oxidative stress can lead to altered glomerular hemodynamics and enhanced glomerular permeability to proteins.^6^ Studies have shown a significant correlation between levels of several oxidative stress parameters and proteinuria in patients with NS.^7^,^8^

Albumin, the most abundant protein in blood, plays an important role in the distribution of a wide range of molecules, including drugs, hormones, and transition metal ions, in organisms. The N-terminal portion of albumin, especially the N–Asp–Ala–His–Lys sequence, is a binding site with high affinity for transition metal ions, such as cobalt, copper, and nickel.^9^ The N-terminal portion of this protein is modified under the conditions of ischemia, hypoxia, acidosis, and increased generation of ROS, and its ability to bind transition metal ions is reduced. This variant form of albumin is called ischemia-modified albumin (IMA), which reflects the cobalt-binding capacity of albumin.^10^ IMA has emerged as a useful biochemical marker in the diagnosis of myocardial ischemia,^11^ and elevated IMA levels are related to a number of clinical conditions, such as type 2 diabetes,^12^ metabolic syndrome,^13^ cancer^14^, and chronic kidney disease.^15^

Since NS is associated with increased oxidative stress, we hypothesized that IMA levels could be elevated in patients with NS. Therefore, in this work, we aimed to evaluate IMA levels as an indicator of oxidative stress in children with steroid-sensitive NS (SSNS) in remission and relapse.

## METHODS

This cross-sectional study was carried out at the Pediatric Nephrology Unit of Sanliurfa Training and Research Hospital, Sanliurfa, Turkey, from April 2019 to December 2019. The study population included 70 children diagnosed with SSNS and 45 healthy controls. Patients were classified into two subgroups: SSNS-remission (n= 50) and SSNS-relapse (n= 20). SSNS remission was defined as nil/trace protein on dipstick or proteinuria <4 mg/m^2^ per hour for three consecutive days. Relapse was defined as 3+ or 4+ protein on dipstick or proteinuria >40 mg/m^2^ per hour for three consecutive days.^1^

Patients with steroid-resistant NS, malignancy, chronic diseases, acute/chronic infection, liver diseases, heart insufficiency, or secondary or congenital NS and those using immunosuppressive drugs were excluded from the study. Patients with NS who received albumin, blood, or antioxidant supplements during the 2-week period prior to the study were also excluded. The study protocol was approved by the Ethics Committee of Harran University (registration number: HRU/18/03/05).

### Biochemical measurements

After overnight fasting, venous blood samples were collected from the patients with SSNS and healthy controls into 5.0 mL BD Vacutainer tubes with gel (Becton, Dickinson and Company, USA). The collected blood samples were centrifuged at 2000 × *g* for 10 min after resting for 30 minutes, and the obtained sera were kept at −80 °C until assayed. Albumin, creatinine, and IMA levels were measured from the serum samples. Serum albumin and creatinine were measured on a Cobas c702 instrument (Roche Diagnostics, Germany) via the bromocresol green and alkaline picrate methods, respectively. IMA levels were determined using the albumin-cobalt binding assay, which measures the binding capacity of the N-terminal region of albumin to exogenous cobalt according to the method described by Bar–Or et al.^11^ Because IMA levels are affected by the albumin concentration, the IMA results were adjusted using the following formula: Adjusted IMA= IMA (ABSU)/albumin (g/dL).^16^

### Statistical analysis

All analyses were carried out using SPPS Version 20.0 (Chicago, IL, USA). The Kolmogorov–Smirnov test was used to check the normality of the variables, and the chi-squared, Mann–Whitney U, one-way ANOVA, or Kruskal–Wallis test (followed by the Bonferroni-corrected Mann-Whitney U test for pairwise comparisons) was used to compare variables. The relationships between IMA and age, disease duration, and albumin were evaluated using Spearman’s correlation coefficient. The levels of significance were set to p<0.017 (0.05/3) for the Bonferroni-corrected tests and p<0.05 for the remaining tests.

## RESULTS

The demographic characteristics and laboratory findings of the study groups are shown in Table-I. No significant differences were observed between the three groups in terms of age, gender, disease duration, and creatinine level *(*p*>*0.05). IMA and adjusted-IMA levels were significantly higher and albumin levels were significantly lower in the SSNS-relapse group than in the SRNS-remission and control groups (for all, p<0.05). Furthermore, IMA and adjusted-IMA levels were significantly higher and albumin levels were significantly lower in the SSNS-remission group than in the control group (for all, p<0.05) (Table-I).

**Table-I:** Demographic and biochemical characteristics of study participants.

Variables	Control group (n =45)	SSNS-remission group (n =50)	SSNS-relapse group (n =20)	Overall P value	Pairwise comparisons
Age (years)	8.9±2.8	9.3±2.6	9.3±3.2	0.767^a^	–
Boy/girl (n)	27/18	32/18	13/7	0.895^b^	–
Disease duration (years)	–	4.2±1.7	4.0±2.0	0.450^c^	–
Creatinine (mg/dL)	0.44±0.09	0.40±0.10	0.44±0.13	0.116^ a^	–
Albumin (g/dL)	4.63 (4.35–4.88)	4.31 (3.59–4.64)	2.82 (1.20–3.36)	<0.001^d^	*P*^1^ = <0.001 *P*^2^= <0.001 *P*^3^= <0.001
IMA (ABSU)	0.67 (0.51–0.79)	0.71 (0.59–1.03)	1.09 (0.84–1.37)	<0.001^d^	*P*^1^ = 0.002 *P*^2^= <0.001 *P*^3^= <0.001
Adjusted-IMA	0.14 (0.11–0.18)	0.17 (0.13–0.27)	0.39 (0.25–1.13)	<0.001^d^	*P*^1^ = <0.001 *P*^2^= <0.001 *P*^3^= <0.001

Significance was accepted at P<0.05. a= One-Way Analysis of Variance test; b= Chi-square test; c= Mann-Whitney U test; d= Kruskal-Wallis test (followed by the Bonferroni-corrected Mann-Whitney U test for pairwise comparisons; *P*<0.017 was considered as significant). P1= Control group vs SSNS-remission group. P2= Control group vs SSNS-relapse group. P3= SSNS-remission group vs SSNS-relapse group.

IMA levels are inversely correlated with albumin levels in patients with SSNS (r= −0.881, p=<0.001) (Table-II). However, IMA levels were not correlated with age or disease duration.

**Table-II:** Association of IMA with age, disease duration and albumin in patients with SSNS.

		Age	Disease duration	Albumin
IMA	*r*	− 0.012	−0.023	−0.881
	*P*	0.923	0.852	<0.001

## DISCUSSION

In the current study, we observed that IMA and adjusted-IMA levels were higher in children with SSNS in remission and relapse than in healthy children. IMA and adjusted-IMA levels were significantly higher in the SSNS-relapse group than in the SSNS-remission group. A negative correlation between IMA and albumin was also observed in all children with NS. To the best of our knowledge, this study is perhaps the first to investigate IMA levels in children with SSNS.

Although NS is a common chronic illness in childhood, its pathogenesis remains incompletely understood. Growing evidence suggests that oxidative stress plays a pivotal role in the pathogenesis of NS.^2^-^4^ Oxidative stress, which is defined as the disruption of balance between ROS and antioxidants, may lead to cellular damage by inducing DNA damage, lipid peroxidation, and protein oxidation.^5^ Exposure of cells to ROS has been suggested to cause impaired tubular epithelial integrity, increased permeability of glomeruli to proteins, and hemodynamic changes in glomeruli.^6^ Oxidative damage to glomerular structures is known to be a major contributor to the course and prognosis of NS.^17^

Albumin, the major plasma protein, is one of the main targets of ROS. Albumin plays a crucial role in antioxidant defense against oxidative attack via various mechanisms, such as transition-metal chelation and free-radical scavenging.^18^ Under ischemic and oxidative conditions, the N-terminal region of albumin may undergo structural changes that decrease the binding capacity of the protein to transition metals, resulting in the formation of IMA.^10^

Since IMA is a practical and inexpensive biomarker that is easily measurable using the albumin-cobalt binding assay^11^, it has been investigated as an alternative indicator of ischemia and oxidative stress in many diseases. Several studies have shown that IMA levels are elevated in ischemic events^11^,^16^,^19^ and oxidative stress-related diseases.^12^-^15^ Nevertheless, the role of IMA has not been examined in patients with NS. In our study, we observed an increase in IMA levels in patients with SSNS in relapse and remission; moreover, this increase was more pronounced in patients with SSNS in relapse than in those in remission. Serum albumin levels significantly decreased in patients with SSNS and showed a negative correlation with IMA levels. IMA levels are well known to be affected by changes in albumin concentration, and an inverse correlation between IMA and albumin levels has been reported.^20^ Therefore, we adjusted the IMA levels of the respondents according to their albumin concentration to interpret the IMA results correctly. We found that adjusted IMA levels were higher in children with SSNS in both relapse and remission groups than in healthy controls and, especially in relapse group, similar to our non-adjusted findings. These results reveal that higher IMA levels observed in NS patients are associated with increased oxidative stress and may indirectly reflect oxidative protein damage. Our results support other investigations showing increased levels of protein oxidation in patients with NS compared with the control group.^21^,^22^ For example, Fan et al.^21^ found higher levels of advanced oxidation protein products and Yazilitas et al.^22^ observed increased thiol oxidation in patients with NS than in their corresponding controls.

Our study also revealed that IMA and adjusted-IMA levels in patients with SSNS in relapse were higher than those in the remission group. These results are in agreement with previous reports showing that the relapse group has more severe oxidative stress than the remission group.^21^-^23^ Furthermore, NS has been shown to be partially related to ischemic conditions, such as pulmonary embolism, deep venous thrombosis, renal vein thrombosis, and mesenteric ischemia results from hyperlipidemia and hypercoagulability states, including increased synthesis of procoagulant factors, urinary loss of anticoagulants, steroid therapy, and platelet hyperaggregability.^24^,^25^ All of these conditions may contribute to the increase in IMA concentration in patients with NS.

### Limitations of the study

Only IMA was evaluated in the study groups. Investigation of other oxidative stress biomarkers and their relationship with IMA is necessary to obtain a comprehensive assessment. In addition, our work included only a small number of participants.

## CONCLUSIONS

We have demonstrated that IMA and adjusted IMA levels are significantly increased in children with SSNS, particularly those in relapse, compared with healthy controls. We have also shown that serum IMA is inversely correlated with albumin. These results suggest that oxidative stress may be related to the pathophysiology of NS. Further studies on IMA in patients with NS should be conducted.

### Authors’ Contribution:

**GC:** Conception & design, acquisition of data, analysis, drafting the article, revision of the article, final approval and responsible and accountable of the study.

**AG, KY & CN:** Acquisition of data, analysis, statistical analysis, revision of the article, final approval.
